# Antiviral Potential of Plants against COVID-19 during Outbreaks—An Update

**DOI:** 10.3390/ijms232113564

**Published:** 2022-11-05

**Authors:** Qazi Mohammad Sajid Jamal

**Affiliations:** Department of Health Informatics, College of Public Health and Health Informatics, Qassim University, Al Bukayriyah 52741, Saudi Arabia; m.quazi@qu.edu.sa

**Keywords:** antiviral, medicinal plants, COVID-19, SARS, MERS

## Abstract

Several human diseases are caused by viruses, including cancer, Type I diabetes, Alzheimer’s disease, and hepatocellular carcinoma. In the past, people have suffered greatly from viral diseases such as polio, mumps, measles, dengue fever, SARS, MERS, AIDS, chikungunya fever, encephalitis, and influenza. Recently, COVID-19 has become a pandemic in most parts of the world. Although vaccines are available to fight the infection, their safety and clinical trial data are still questionable. Social distancing, isolation, the use of sanitizer, and personal productive strategies have been implemented to prevent the spread of the virus. Moreover, the search for a potential therapeutic molecule is ongoing. Based on experiences with outbreaks of SARS and MERS, many research studies reveal the potential of medicinal herbs/plants or chemical compounds extracted from them to counteract the effects of these viral diseases. COVID-19′s current status includes a decrease in infection rates as a result of large-scale vaccination program implementation by several countries. But it is still very close and needs to boost people’s natural immunity in a cost-effective way through phytomedicines because many underdeveloped countries do not have their own vaccination facilities. In this article, phytomedicines as plant parts or plant-derived metabolites that can affect the entry of a virus or its infectiousness inside hosts are described. Finally, it is concluded that the therapeutic potential of medicinal plants must be analyzed and evaluated entirely in the control of COVID-19 in cases of uncontrollable SARS infection.

## 1. Introduction

Several human diseases are caused by viruses, including cancer, Type I diabetes, Alzheimer’s disease, and hepatocellular carcinoma. In the past, people have greatly suffered from viral diseases such as polio, mumps, measles, dengue fever, SARS, MERS, AIDS, chikungunya fever, encephalitis, and influenza. Human cancer viruses include the hepatitis B virus, the Epstein-Barr virus, the hepatitis C virus, the human T-cell lymphotropic virus type 1, high-risk human papilloma viruses, and Kaposi’s sarcoma-associated herpesvirus [1]. Human enterovirus (HEV) has long been thought to act as an environmental trigger for the onset of Type 1 diabetes (T1D) in people [2]. HHV-6A and HHV-7, two human herpesviruses, may be major causes of AD; however, HSV infection with other viral infections has been documented in some of these cases [3]. The disease 2019-nCoV originated in Wuhan, China, at the end of December 2019, and the WHO declared it an international emergency with public health concerns and issued International Health Regulations [4]. The disease is a pandemic and ongoing; therefore, it is critical to search for new preventive and therapeutic methods as soon as possible. The virus causing 2019-nCov was identified as a β-coronavirus, specifically severe acute respiratory syndrome virus 2 (SARS-CoV-2), belonging to the coronavirus family. COVID-19 is characterized by a series of complex clinical symptoms, including fever, pneumonia, dry cough, and shortness of breath. As of 27 July 2022, the World Health Organization announced 570,005,017 confirmed cases and 6,384,128 deaths worldwide. Few infected people were treated, and few treatments are currently available [5,6].

**Figure 1:**
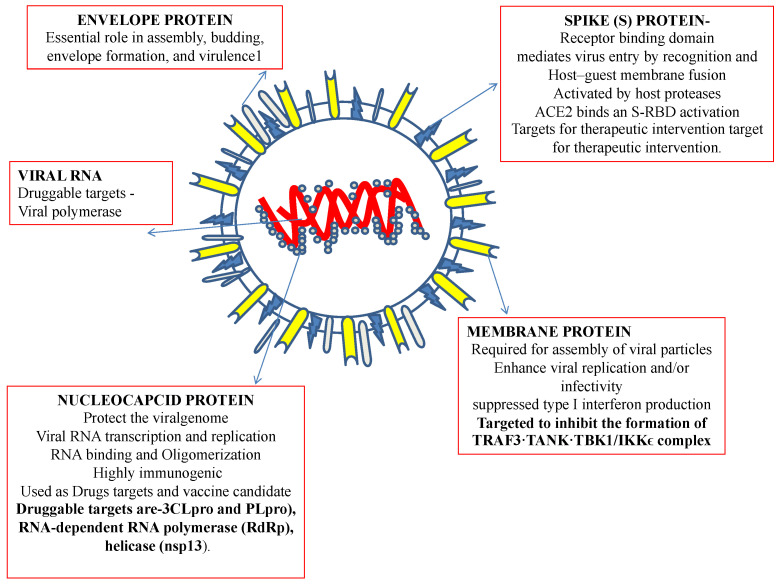
Structural features of a coronavirus.

Recently, an immunization program has led to extensive successful outcomes. However, since its emergence, SARS-CoV-2, the virus causing COVID-19, has challenged public health. Recently, an outbreak of COVID-19 has driven the focus toward traditional herbs that are available in different parts of the world. Many therapeutic and diagnostic strategies are available to manage the disease [7,8]. Moreover, studies have reported that SARS-CoV-2 infection can be spread to reptiles, avian species, and mammals [9]. At least 21 families of viruses can cause disease in humans. Of these, five viral families consist of dsDNA, three families comprise nonenveloped viruses (Adenoviridae, Papillomaviridae, and Polyomaviridae), and two families comprise enveloped viruses (Herpesviridae and Poxviridae). One family consists of partially double-stranded (ds) DNA (Hepadnaviridae and enveloped viruses). The members of seven families are single-stranded RNAs, of which three families are characterized as nonenveloped (Astroviridae, Caliciviridae, and Picornaviridae), and the members of four families are enveloped (Retroviridae, Coronaviridae, and Flaviviridae). All nonenveloped families carry icosahedral nucleocapsids and are in negative ssRNA families (Arenaviridae, Bunyavirales, Filoviridae, Orthomyxoviridae, Paramyxoviridae, and Rhabdoviridae) and are enveloped with helical nucleocapsids. One virus, a dsRNA (Reoviridae, which causes hepatitis D), has not been assigned to a family [8,9]. SARS-CoV-2, the virus causing COVID-19 has been identified as an RNA virus with a special spike protein, nucleocapsid protein, membrane protein, envelope protein, and enzymatic proteins, which have been reported to be therapeutic targets that control disease (Figure 1). Secondary metabolites are synthesized by medicinal plants for plant defense. Many therapeutic molecules, such as antibacterial, antifungal, and antiviral molecules, have been isolated, purified, characterized, and used in the management of various diseases.

To develop medicines against viruses, including SARS-CoV-2, we must focus on these secondary metabolites. Among secondary metabolites, flavonoids have been reported to exhibit antiviral properties [10]. Certain medicinal plants and their respective antiviral potential are described in Table S1. However, thorough guidance for employing several traditional medicines to treat viral infections, including coronaviruses, has not been offered, and the mechanisms of virus action have not yet been fully explained. Notably, more plants or herbs can be considered as therapeutics to increase the supply of medicinal components with activity against viruses. The majority of previous papers and studies have primarily concentrated on specific ethnobotanical drugs, such as traditional Chinese or Indian medicines [11,12,13,14,15], without regard to particular locations or ethnobotanical characteristics. In addition to core wet laboratory experiments, several modern computational methodologies have been adopted for screening and identifying natural compounds that may inhibit viral infection, and the utilization of these approaches to identify fast-acting and more potent vaccines and treatments, including drugs, for COVID-19, increased during the recent pandemic [16,17,18]. Many major scientific databases have been established to help researchers identify possible ethnomedicinal plants or herbs for use as antivirals, including for coronavirus treatments. All previously published reports can be found using the search terms medicinal plants, herb OR herbal, (virus OR viral), and COVID-19/corona in PubMed, Scopus, Web of Science databases, and in Google Scholar, among other online resources. We searched for, found, and thus included studies from these databases in this review. Relevant articles were chosen after being critically assessed for their usefulness; they are described on the basis of the research described, that is, on whether experiments were performed in silico, in vitro, or in vivo [11].

## 2. Therapeutic Targets for Coronaviruses

Human coronavirus was first detected in the 1960s and was broadly classified into four categories, i.e., alpha, beta, gamma, and delta. There are currently seven known coronaviruses that can harm humans, namely, 229E (alpha coronavirus), NL63 (alpha coronavirus), OC43 (beta coronavirus), HKU1 (beta coronavirus), MERS-CoV (the beta coronavirus that causes Middle East Respiratory Syndrome, or MERS), SARS-CoV (the beta coronavirus that causes severe acute respiratory syndrome, or SARS), and SARS [19].

Molecular level investigation and characterization of the SARS-CoV-2 genome is almost 80% similar to the genetic content of SARS-CoV-2 with characteristics 10b, 13, and 14 different gene regions [20].

SARS-CoV and SARS-CoV-2 viruses use human ACE2 as an entry receptor and human proteases as entry activators, which differentiate from each other [21,22]. Moreover, the most important structural protein, i.e., the spike protein (S), is slightly different in these viruses. SARS-CoV-2 has a furin-like cleavage site that makes the S protein priming easier and may make it more effective at spreading than other beta coronaviruses. Furin inhibitors are thus a possible target for SARS-CoV pharmacological treatments [23,24].

However, both viruses utilize a spike receptor-binding domain for recognition and host cell infection with the support of cellular serine proteases [22].

Currently, no trusted therapeutic intervention is available to target and manage COVID-19. For the development of effective therapy against any disease, the drug-targeting sites or cell-binding proteins must be identified. Recently, the COVID-19 virus genome and potential cell-binding proteins have been discussed, which will help in the design of therapeutics and treatment strategies for this disease (Figure 1).

### 2.1. Spike Protein

It has been reported that an anthraquinone glycoside similar to emodin, which had been previously found encoded in *Polygonum* and Rheum genera, blocks the interaction between the host ACE2 and viral S protein in a dose-dependent manner. The spike protein is a Type-1 trimeric crown-like protein (Type I-TM protein) that facilitates virus entry by binding with the receptor ACE-2 (the receptor of DPP-4) mediated via the viral S1 unit and the host enzyme cathepsin L. Spike is a three-segmented protein; that is, it comprises an ectodomain segment (ED), TM segment, and intracellular domain. The ectodomain forms three S1 heads, which are called receptor-binding domains S1, and at the C-terminus, a trimeric stalk is known as the S2 subunit, which fuses to the cell membrane Importantly, SARS-CoV binding to its host cell receptor (ACE2) seems to be necessary for SARS-CoV-2 entry into cells. Viral RNA enters host cells and mediates the progression of the disease. Moreover, spike proteins activate the immunological response of the host cell to viral entry. Therefore, spike protein components are essential targets for drug discovery research [25]. Many plant metabolites, such as quercetin and kaempferol, have been used to block spike-mediated viral infection [26]. Recently, Magdalena reported that oral administration of the coronavirus spike protein confers protection to newborn pigs when challenged with a porcine epidemic-causing diarrhea virus. Magdalena claimed that when neonatal piglets were exposed to a swine-epidemic-causing diarrhea virus, oral treatment with the coronavirus spike protein protected them from infection [27].

### 2.2. Envelope Protein

The smallest envelope protein has a molecular weight of 8.4–12 kDa. It is made up of a hydrophobic domain and a charged cytoplasmic tail. It plays a significant role in modulating the activity of intracellular proteins, ion channel formation, and morphogenesis, particularly during viral efflux and assembly. Moreover, it has also been described as a virulence factor. The envelope protein is an essential membrane protein that is necessary for the assembly, morphogenesis, and discharge of a virus within the host. Hexamethylene amiloride has been reported to function as an E protein-associated ion channel [28]. Oedema and other ARDS-specific symptoms are caused by the migration of syntenin from the nucleus to the cytoplasm, which is mediated by the E protein, activating p38 MAPK and leading to the synthesis and secretion of proinflammatory cytokines such as IL-1 [29]. To date, only five host proteins have been identified as interactors of the E protein: Syntenin, sodium/potassium (Na+/K+) ATPase-1 subunit, stomatin, and PALS1 are three of these five molecules. The versatile SARS-CoV-2 envelope (E) protein aids in viral replication and efflux as it is a membrane protein with viroporin-like performance parameters that may contribute to disease etiology, illness severity, and disruption of the epithelial barrier. A putative PDZ-domain binding motif (PBM) has also been found in the extensive C-terminal (ECT) region of E, including in the E protein of SARS-CoV-1 [30].

### 2.3. Membrane Protein

The membrane protein plays a vital role in viral envelope shape maintained by M- and N-type proteins, nucleocapsid protein stability, and viral intracellular homeostasis. The viral membrane protein comprises three domains, with the C-terminal projected inside of the virus and the short N-terminal domain projecting outwards. Viral assembly is due to M–M (host–virus), M–S (spike protein–Golgi complex interaction), and M–N (nucleocapsid–RNA complex) types of interactions. The stabilization of host cells and the activation of IFN-beta and the nuclear factor-kappa pathway, particularly in SARS-CoV infection, are functions of the viral M protein [30,31,32]. Recently, the SARS-CoV-2 membrane protein has been shown to block the expression of Type I interferons by degrading TBK1 via ubiquitin-mediated mechanisms.

The Pangolin CoV MP798 isolate and Bat CoV isolates contain identical envelope proteins. Deletions at amino acid position 70, corresponding to a Gly or Cys residue in other envelope proteins, and the replacement of an Arg residue with a Glu residue, make these two proteins unique. Comparisons of membrane glycoproteins have indicated that those of SARS-CoV are significantly different from those of other viruses. The peptides in the envelope, however, are comparable. Impactful mutations in the envelope protein may greatly change E-protein structural characteristics and perhaps protein-protein molecular interactions. Variations in the membrane protein, which functions with the spike protein during viral adhesion and uptake by cells, may also be important. Therefore, mutations to the E protein might exert an impact on how coronaviruses interact with host cells [33].

### 2.4. Nucleocapsid Protein

Nucleocapsid proteins or N proteins have been reported to be conserved in different members of the CoV family. The protein is characterized by the presence of an N-arm, central linker (CL), enriched with phosphorylation sites, serine and arginine, and a C-terminal tail. The NTD structure is essential for functional RNA-binding and is involved in CTD-driven dimerization. Moreover, the N protein has been reported to regulate the replication and transcription of viral RNA, even in host cells. However, it inhibits translation upon association with EF1α [34,35]. Thus, it has been reported that the endoplasmic reticulum–Golgi intermediate compartment (ERGIC), where the M protein is located after synthesis in the cells, serves as a platform for attracting other viral structural proteins10. At a minimum, the combination of M + N is necessary for the production of virus-like particles (VLPs) when SARS-CoV-2-specific proteins are co-expressed in mammalian cells. This finding demonstrates an essential function of the M protein in the assembly of viruses. For SARS-CoV and the mouse hepatitis virus (MHV) 14, another coronavirus, to properly form viral particles, the M and N proteins must be paired with the appropriate host cell types. Additionally, relationships between the M and N proteins of SARS-CoV-215, as well as the M protein and genomic RNA carrying the MHV16 packing signal, have been documented. These findings on SARS-CoV-2 and related viruses imply that the M protein plays various important roles in coronavirus assembly.

### 2.5. Proteases

The genome of SERS-CoV synthesizes various proteins, including enzymes. The replica gene, which is a key part of the CoV virus genome, encodes 16 NSPs, which are large PP (PP1a and PP1ab) proteins. After release from the C-termini of these PPs, the NSPs are processed by a 3C-like protease [3CLpro]) or the main protease [Mpro], a cysteine protease chymotrypsin, while cleaved N-terminal peptides are processed through the Mopar or papain-like protease [PLpro]) [36]. The papain-like protease PLpro cleaves an N-terminal PPs, generating NSPs 1, NSPs 2, and NSPs 3 and replicate substrates. The cleavage process requires the LXGG consensus sequence. The PLpro and CLpro proteases can be inhibited via zinc or zinc conjugates. Haemagglutinin esterase (HE) is a marker enzyme of influenza and beta-coronaviruses in the viral envelope. Esterases facilitate the reversible binding t O-acetylated sialic acids by acting as both receptor-damaging and lectin-damaging enzymes. NTPase/helicase is a member of the SF1 helicase family that makes a key contribution to the central dogma of viral infection. It hydrolyses NTPs and utilizes dATP, ATP, and dCTP substrates [37].

### 2.6. Endosomal pH

The absorption, distribution, metabolism, and even excretion of many drugs and therapeutic molecules depend on pH. Moreover, the entry of many microbes is facilitated by a particular pH environment. A low pH favors the successful progression of the coronavirus life cycle. Molecules that inhibit pH-dependent endosomal proteases may be useful in managing viral infection. A low pH also favors the accumulation of amiodarone, which results in alterations to endosomes and inhibits coronavirus infection [38].

## 3. Antiviral Potential of Plant Extracts/Metabolites for Treating SARS-CoV-2 Infection

As COVID-19 rapidly spread, the search for natural antiviral molecules that can fight a SARS-CoV-2 infection was accelerated. Several in silico and in vitro/in vivo studies on plants as well as repurposed drugs have been conducted to identify potential therapeutic molecules. For a significant effect, the binding interactions must be facilitated between ligand and drug molecules. The SARS-CoV-2 infection has also been studied for evaluating drug targets such as spike protein, envelope protein, membrane protein, nucleocapsid protein, and proteases, using various cellular and animal models. Although in vitro studies are helpful for understanding virus biology in highly controlled environments, these models frequently fall short of accurately recapturing the complexity of true bodily systems. However, in vivo research is expensive, requires BSL-3 animal facilities, and raises ethics questions. Two-dimensional (2D) or three-dimensional (3D) cultivation of immortalized cells or primary cells and tissues serves as the foundation for in vitro models [39]. Recently, hydroxychloroquine and chloroquine, and glycyrrhizin, have been evaluated through in vitro studies. turmeric (*Curcuma longa* L.; Zingiberaceae.) rhizomes, mustard (*Brassica nigra* W.D.J. Koch; Brassicaceae), and wall rocket (*Diplotaxis erucoides* subsp. *erucoides*; Brassicaceae), were reported to considerably suppress 3CLPro activity when administered at 500 g mL^−1^ [40].

**Figure 2:**
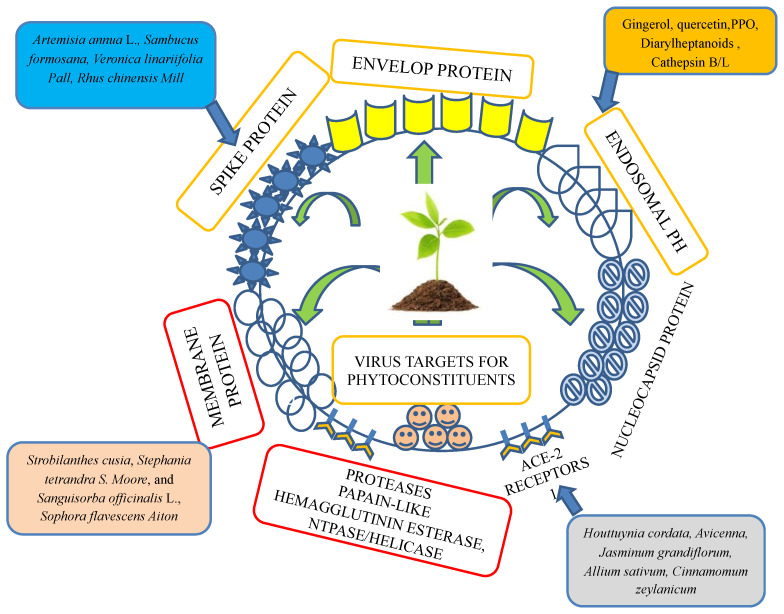
Plant- and herb-targeted envelope protein, spike protein, membrane protein, protease inhibitors, ACE-2 receptors, nuclear capsid protein, and endosomal-like parts of a virus.

A study proposed that a potential approach involves garlic essential oil. The active molecule in this essential oil simultaneously inhibited ACE-2 protein activity, leading to a loss of this viral receptor in the host cell, and attacked the receptor with PDB ID 6LU7 (the main protease in SARS-CoV-2) (Figure 2). A previous docking study showed that 17 chemical components from 18 chemical constituents of oils inhibited ACE-2 protein–virus binding, and these 17 compounds account for 99.4% of all essential oils [41,42]. Many chemicals extracted from traditional herbal spices, such as allicin, E/Z-ajoene, allin, Diallyl disulfide, Diallyl trisulfide, pyrogallol, protocatechuic acid, quercetin, and gallic acid from the *Allium cepa* L. bulb (Amaryllidaceae), have been reported to show significant antiviral and antibacterial activities, which could also be active against SARS-CoV-2. Moreover, Singh et al. discussed its antimicrobial potential [43].

*Piper longum* L. Piperaceae, a fruit commonly known as Indian spice kali mirch, has been reported to show antiviral activity against Coxsackie virus type 3 (CVB3) due to the presence of α-pinene, β-pinene, limonene, myrcene, sabinene, camphene, α-thujone, piperitone, caryophyllene, p-cymene, α-terpinene, and piperamide [44]. A study on curcumin described its inhibitory action on viruses in addition to SARS-CoV-2. *Curcuma longa* L. (Zingiberaceae) is enriched with curcumenone, bisacumol, bisacurone, curcumenol, curcumadiol, and demethoxycurcumin. Curcumin inhibits SARS-CoV-2 replication in human cells, as previously reported for HIV-AIDS [44,45], chikungunya virus, Zika virus, and herpes simplex virus (HSV).

Moreover, curcumin has been reported to inhibit the penetration of SARS-CoV-2 into host cells to prevent infection. The prevention of chikungunya and Zika virus infectivity by curcumin has been reported, and this feature of turmeric may be beneficial for the therapeutic inactivation of COVID-19 [46].

*Syzygium aromaticum* (L.) Merr. & L.M. Perry (Myrtaceae) flowering buds have been reported to contain eugenol, acetyl eugenol, β-caryophyllene, vanillin, eugenin, kaempferol, rhamnetin, and eugenitin, and they have antiviral potential against SARS-CoV-2, herpes simplex virus I, herpes simplex virus 2, and the hepatitis C virus.

A docking study reported that eugenol shows low binding energies with viral proteins; for example, its binding energies were −6.1 and −5.4 kcal/mol for the S protein (PDB ID 6VXX) and M^pro^ (PDB ID 6LU7), respectively [47]. However, this low binding energy showed a less significant effect than the even lower binding energies of nelfinavir with these proteins, which were −8.8 and −8.2 kcal/mol, respectively.

Sage essential oils as phytomedicines have recently been discussed, particularly as antiseptics and sanitizers. The essential oil in *Salvia officinalis* L. has been reported to exert an effect against SARS-CoV in a patient at the Frankfurt University Hospital, showing an IC50 = 870 mg/mL, which was low [48,49]. The main chemical compounds in the essential oil extracted from *Laurus nobilis* L. that have been reported to inhibit SARS-CoV and HSV-1 replication were 1,8-cineole, beta-ocimene, beta-pinene and alpha-pinene, each with t an IC(50) value of 120 µg/mL [50]. Thus, it can be concluded from previously conducted research that essential oils have limited antiviral activities against viruses, including SARS-CoV-2 [48]. Thus, symptomatic alleviation after infection may be achieved by using essential oils such as eucalyptus oil, eugenol, cinnamon oils, and neem oil.

*Zingiber officinale Roscoe* from Zingiberaceae and rhizome have been reported to contain therapeutic chemicals, including 6-gingerol, 6-shogaol, 6-paradol, zingerol, and gingerol, with activity against SARS-CoV-2 and human respiratory syncytial virus (HSRV). The antiviral potential of *Zingiber officinale Roscoe* against viruses, including SARS-CoV-2, has been described on the basis of computational approaches. The results of docking studies have indicated that 6-Sogaol showed binding energies of −5.5 and −5.8 kcal/mol to the S protein (PDB ID 6VXX) and M^pro^ (PDB ID 6LU7), which were comparable to the binding energies of nelfinavir for these proteins (−8.8 and −8.2 kcal/mol, respectively). In silico studies indicated that eight chemicals extracted from rhizomes of *Alpinia officinarum* Hance, Zingiberaceae, and gingerol were potential protease (PLpro) inhibitors of SARS-CoV-2. Thus, the aforementioned studies showed that phytoconstituents or their extracts were SARS-CoV-2 inhibitors, suggesting that these compounds are promising therapeutic molecules against SARS-CoV-2 [51,52]. The role of *Schizachyrium urceolatum* Stapf Poaceae as an antioxidant suggests that cinnamon shows appreciable immunostimulatory activity by increasing phagocytic activity [53].

The root and flower of clove pink, also known as *Dianthus caryophyllus* L., has been reported to contain dianthin30, dianthin32, dianthramides, flavonoids against SARS, herpes simplex virus-I (HSV-I), hepatitis-A Virus-27 (HSV-27) [45]. The antiviral activity against herpes simplex virus-I and hepatitis A virus-27 of crude seed extract are found in this plant. At a nontoxic concentration (20 µg/mL), the extract applied to both Vero and HepG-2 cells led to potent antiviral effects on HSV-I and HAV-27, as determined using a plaque infectivity count assay [45]. *Dianthus caryophyllus* L. as reported to exhibit antiviral activity against HSV-I and HAV-27, so this plant could also be a potential source for antiviral activity against SARS-CoV-2.

The *Phytolacca americana* L. Phytolaccaceae plant synthesizes ribosome-inactivating protein (RIP). A single-chain ribosome-inactivating protein has been analyzed. Studies provided evidence showing that exogenous application of RIP inhibits zucchini yellow mosaic virus (ZYMV) infection in squash plants in a concentration-dependent manner [54]. The antiviral activity of the *Mirabilis jalapa* L. plant contains 4-hydroxycoumarin, mirabijalones A-D, and 9-o-methyl-4-hydroxyboeravinone B. The root extract of these compounds inhibits the multiplication of tobacco mosaic virus via the inactivation of ribosomes that had been stimulated by the virus [55]. The antiviral effect of *Camellia sinensis* (L.) Kuntze extract, also known as green tea, has been reported to inhibit the expression of hepatitis surface antigen (HBsAg) and hepatitis B antigen (HbeAg). Studies reported that the extract exerted an inhibitory effect on intestinal α-glycosidases that are important for processing glycoproteins and glycolipids in viruses [51,56,57]. Kim et al. reported that the methanolic extracts of *Acanthopanax* (Decne. & Planch.) Miq. Araliaceae and *Cimicifuga acemose* (L.) Nutt. were administered at doses of 0.9 ± 0.1 μg/mL and 19.4 ± 7.0 μg/mL, inhibiting MHV-A59-type coronavirus, respectively. The IC_50_ of the ethanolic extract of *Artemisia annua* L. against SARS-CoV BJ-001 was 34.5 ± 2.6 μg/mL [58,59]. *Euphorbia neriifolia* L., Euphorbiaceae enriched with 3-β-friedelanol, 3-β-acetoxy friedelane, friedelin, and epitaraxerol-like chemical compounds has also been described to significantly inhibit HCoV [60]. The IC50 of an aqueous extract with 191.6 ± 8.2 μg/mL of *Isatis indigotica* Fortune ex Lindl; Brassicaceae and other bioactive molecules, e.g., 752 μM Indigo, 217 μM Sinigrin, 1210 μM beta-sitosterol, 366 μM aloe emodin, and 8.3 μM Hesperetin, indicated the antiviral potentiality of this plants against SARS-CoV. Among the compounds from this plant that were tested, low-dose hesperetin showed the greatest antiviral molecule activity. The aquas extract of *Polygonum multiflorum* Thunb. Polygonaceae (root/vine) has been reported to exhibit significant antiviral activity (IC50 1–10 μg/mL) against SARS-CoV [52]. Chen et al. reported the antiviral effect of a water-based extract of *Toona sinensis* (Juss.) against SARS-CoV, which showed efficacy at an IC50 of 30–43 μg/mL [61]. When a 6% Epimedium aqueous extract was administered in the test model it led to no diarrhoeal symptoms [62], and intestinal biopsy sample assays revealed complete eradication of the virus from the intestine of the tested animals. The roots of *Polypodium glycyrrhiza* D.C.Eaton (Licorice) have been used as a traditional medicine for bronchitis, peptic ulcers, allergies, inflammation, and asthma for a long time. Licorice root has been known to be a powerful viral infection inhibitor since ancient times [63,64,65,66]. Glycyrrhizic acid, also referred to as glycyrrhizin, is a key chemical compound in the triterpenoid glycoside class of herbs. SARS-CoV has reportedly been suppressed with glycyrrhizin [67]. Replication of clinical isolates of SARS-CoV have been reportedly inhibited by glycyrrhizin more effectively than a number of synthetic antivirals, including mycophenolic acid, pyrazofurin, 6-azauridine, and ribavirin [68]. Many plants and foods contain high levels of the flavonoid quercetin, which shows a wide range of therapeutic and pharmaceutical effects [69]. Both quercetin 3-galactoside and quercetin abrogate the SARS-CoV 3CLpro protease function in vitro [70].

Wen et al. discovered that specific lignoids and abietane-type diterpenoids show the strongest SARS-CoV-2 inhibiting activities, and they are present in *Cryptomeria japonica* (Thunb. ex L.f.) D.Don; Cupressaceae *Juniperus formosana* Hayata Cupressaceae, and *Chamaecyparis obtusa* Siebold & Zucc. Cupressaceae [71]. Moreover, other bioactive substances related to the aforementioned compound classes are present in *Cibotium barometz* (L.) J.Sm. Cibotiaceae (dried rhizome), *Gentiana scabra* Bunge, Gentianaceae (dried rhizome), *Dioscorea batatas* Decne., Dioscoreaceae (tuber), *Taxillus chinensis* (DC.) Danser, Loranthaceae (leaf), and *Cassia tora* L. Fabaceae (dried seed), which explains the anti-SARS-CoV potential confirmed through a cell-based assay with infected Vero E6 cells at concentrations between 25 [71]. Through their potential anti-inflammatory molecules, *Boswellia serrata* extracts may be promising compounds for the management of inflammatory complications during COVID-19 [72].

The antiviral effect of *Bupleurum falcatum* subsp. *cernuum* (Ten.) Arcang; Apiaceae (*Radix bupleuri)* has been reported to explain the presence of saikosaponins A, B2, C, and D. These active constituents are naturally derived triterpenoid glycosides recovered from the *Radix bupleuri* plant. By targeting viruses, these compounds effectively prevent the early stage of HCoV-22E9 infection after facilitating the entry of the virus through viral attachment to the host cell [67,73,74].

Clinical studies on *Glycyrrhiza glabra* L. (Fabaceae) against coronavirus revealed that glycyrrhizin controlled viral SARS-CoV replication. A comparative study was performed using ribavirin, 6-aziridine, pyrazofurin, mycophenolic acid, and glycyrrhizin, to analyze their antiviral activities against coronaviruses (FFM-1 and FFM-2) obtained from clinical patients suffering from SARS. At the end of the study, it was demonstrated that glycyrrhizin at nontoxic concentrations inhibited the replication of SARS-CV; its selectivity index was 67 and its IC50 was 300 mg/mL [68,75]. Glycyrrhizin derivatives, such as glycyrrhetinic acid, derivative GL 1, derivative GL 3, derivative GL 9, derivative GL 10, derivative GL11, derivative GL, and derivative GL 13, from *Myosotis radix-palaris* A.P.Khokhr; Boraginaceae (*Radix scutellariae)*, are traditional Chinese medicines. The active constituent of this plant is baicalin. In a molecular docking study, baicalin exhibited a binding energy of −8.46 kcal/mol, which was sufficient to bind the receptor ACE-2 in the host cell; thus, baicalin may be a potential candidate for 2019-nCoV treatment. Recently, in vitro antiviral activity against SARS-CoV-2 3CLpro, causing pandemic disease, showed that *Scutellaria baicalensis* potentially inhibited the virus with an EC50 of 0.74 µg/mL [76,77]. This citrus plant is abundant in China and has been reported to exhibit anti-SARS activity. Another Chinese herb with reported antiviral activity is *Houttuynia cordata* Thunb.; Saururaceae, which inhibited viral protease 3CL activity and blocked the activity of viral enzyme RNA-dependent RNA polymerase [78,79]. Similarly, *Rheum* and *Polygonum* spp. inhibited hepatitis B virus (HBV) in vitro [79]. The papain-like protease (PL^pro^), which controls the replication of SARS-CoV, has been described as a potential therapeutic target. Diarylheptanoids isolated from *Alnus japonica* Siebold & Zucc.; Betulaceae showed potent (PL^pro^) activity, with an inhibitory concentration (IC_50_) value of 4.1 µM. *Oleo europaea* has also been shown to be a natural antioxidant [80,81,82].

Thus, the aforementioned compounds clearly indicate the potential of plants/herbs in controlling the disease. Although their compounds are not magic potions to treat disease, they may reduce discomfort and perhaps enhance patients’ overall wellbeing. Only extremely chemically well-characterized and pharmacologically well-researched high-quality preparations are suitable for use as herbal remedies that can be considered medicines. Thorough characterization of formulations in future pharmacological and clinical research is crucial [83].

### 3.1. Plant/Herb Targeting ACE

**Figure 3:**
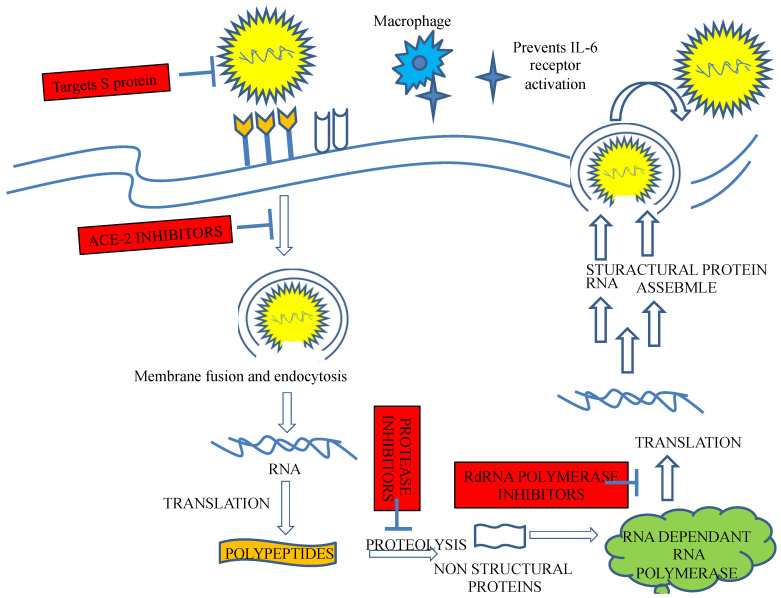
Possible beneficial targets in ACE2-mediated SARS-CoV-2 infection. SARS-CoV-2 binds the cell receptor ACE-2 to facilitate entry after the spike protein is primed by membrane protease. The virus then replicates abundantly, producing replicates that efflux from host cells, resulting in damage and spreading. Potential therapeutic approaches include the use of a soluble form of ACE2 that competitively binds viruses.

The antihypertensive roles of various medicinal plants have been extensively explored. More than 74 families of plants that exhibit significant antihypertensive activities through ACE inhibition have been reported in various scientific studies. Moreover, more than 16 plant families exhibited an antihypertensive effect by blocking AT1 receptor activity, according to in vitro studies [84]. The pharmacological activities, including the antiviral effect of *Avicennia* L.; Acanthaceae, *Jasminum grandiflorum* L.; Oleaceae, *Allium sativum* L.; Amaryllidacea, *Cinnamomum zeylanicum* Blume; Lauraceae, *Vaccinium myrtillus* L.; Ericaceae, *Tribulus terrestris* L. (Zygophyllaceae), and *Vitis vinifera* L. from family Vitaceae, have been extensively explored. Recently, the potential of angiotensin-converting enzyme 2 in the management of severe acute respiratory SARS-CoV-2 has been described. Molecular and phylogenetic studies suggested that similar to other viruses, SARS-CoV-2 binds cell receptor ACE2 to facilitate entry into cells and, therefore, blocks this receptor and may be an effective way to manage serious outbreaks. Moreover, ACE inhibitors, including ACE2 inhibitors, are commonly used to treat cardiovascular complications. ACE2 and the renin-angiotensin system (RAS) exert protective effects on clinical manifestations of diseases, thereby maintaining, for example, fluid and electrolyte balance and cardiovascular homeostasis [21,84]. Hence, the deregulation of the ACE2 function results in a loss of the protective effect of the RAS (Figure 3). Therefore, many phytomedicines that maintain the balance of the normal physiological conditions of cells have been described. More information on ACE2 obtained from outbreaks of SARS-CoV-2 and other SARS infections may provide useful evidence for epidemic preparedness, which is necessary due to their inherent devastation [21,84].

The inhibitory activity of *Nicotiana* spp. on the receptor ACE-2 has been explored, and soybean and tobacco plants were reported to be enriched with nicotianamine, an ACE-2 protein inhibitor [21,85]. The results of a previously conducted molecular docking study indicated that this inhibitor has the potential to bind the receptor ACE-2 with an estimated binding energy equal to −5.1 kcal/mol. Since receptor ACE-2 is critical for 2019-nCoV infection, it may be hypothesized that nicotianamine can block 2019-nCoV infection.

The inhibitory potential of the Chinese herbs in the *Magnolia* genera, such as *Magnolia liliiflora* and *Lonicera japonica*, on ACE enzyme activity has been reported. The active chemicals were reported to be caffeoylquinates (methyl 3,4/5-di-O-caffeoylquinate), catechin-3-O-gallate, flavan-3-ols gallocatechin, epigallocatechin-3-O-gallate, epicatechin-3-O-gallate, and gallotannins epigallaocatechin-3-O-methylgallate, each with an IC50 < 200 AM. The inhibition of ACE activity by caffeoylquinates has been reported to be mediated through chelation with an ACE zinc cofactor. However, other tannins, such as epigallocatechin-3-O-methylgallate, gallotannin: 1, 2, 3, 4,6-penta-O-galloyl-h-D-glucose, and epigallocatechin-3-O-gallate, have been reported to show nonspecific inhibitory effects [86,87]. Methanolic extracts of *Salvia acetabulosa* L. (Lamiaceae) and *Marrubium radiatum* Delile ex Benth. (Lamiaceae) inhibited amylase, glucosidase, and angiotensin convertase enzymes at IC50 values of 52.7 μg/mL and 72.7 μg/mL, respectively. *Salvia acetabulosa* L. exhibited high inhibitory activity and may be another choice for the management of COVID-19 [88].

Root tubers of certain members in the family Polygonaceae, such as the *Polygonum multiflorum* thunb (*Radix polygoni multiflori*), *Rheum officinale* Baill (*Radix et Rhizoma Rhei*) and the vines of *P. multiflorum* Thunb (*Caulis polygoni multiflori*), have been reported to stop the interaction between the host ACE2 and SARS-CoV S proteins at therapeutic IC50 values ranging from 1–10 μg/mL. Moreover, emodin-like anthraquinone glycosides recovered from *Polygonum* and Rheum species have been reported to block the interaction between the ACE2 and S proteins in a dose-dependent manner. Thus, we suggest that emodin may be regarded as a lead therapeutic molecule for the management of COVID-19, in addition to other SARS infections [52]. The IC_50_ of the traditional medicinal plant Persia and the bulb of *Allium sativum* L. (Amaryllidaceae) was 58%: 0.3 mg/mL and 87%: 0.2 mg/mL, respectively.

*Cinnamomum zeylanicum* Blume (Lauraceae) has been reported to contain chemical compounds such as cinnamaldehyde, eugenol, cinnamic acid, cinnamyl acetate, cinnamyl alcohol, and thujene. *Cinnamomum zeylanicum,* aerial *Jasminum grandiflorum* L. exhibited an IC50: 78%; 0.33 mg/mL, and aerial *Tribulus terrestris* (Lamiales) exhibited an IC50 of 0.33, similar to that in the leaf of *Vaccinium myrtillus* L. (Ericaceae) and the fruit of *Vitis vinifera* L. (Vitaceae) Moreover, it has been reported that jasmine inhibited ACE activity at IC50 values of 26–36 μM, secoiridoid aglycones from *jasmine* exhibited IC50 values of 20–25 µM, and sambacein I–III of *J. grandiflorum* each exhibited an IC50 value of 30 μM.

Aqueous leaf extracts, methanolic extracts, and ethanolic extracts from *Azadirachta indica* A.Juss, (Meliaceae), (Fabaceae), *Pongamia pinnata* (L.) Merr., (Fabaceae) and *Catharanthus roseus* (L.) G.Don (Apocynaceae), and *Tamarindus indica* L. (Fabaceae) seed coat extracts reportedly showed higher potential ACE inhibition than their acetone extract counterparts. The pharmaceutical significance of the herb *Peperomia pellucida* has been described; it produces novel pellucidin A and 2,3,5-trimethoxy-9-(12,14,15-trimethoxybenzyl)-1H-indene molecules with potential ACE inhibitory activity [IC50 values of 4.4 µg/mL (11 µM) and 27.95 µg/mL (72 µM), respectively]. The ACE inhibitory potential of betulinic acid (IC50 of 26.77 μM) and piceatannol (IC50 of 8.44 μM) recovered from *Senna garrettiana* (Craib) H.S.Irwin (Fabaceae) & *Chamaecrista rondacorensis* (H.S.Irwin & Barneby) H.S.Irwin & Barneby (Caesalpiniaceae) has also been reported [84,89].

### 3.2. Targeting the S Protein

The G receptor-binding domain in the S1 subunit of the S protein of coronaviruses, including that in SARS-CoV-2, is essential for viral adsorption, as it integrates with S2 via ACE receptor, and penetrates into the host cell. Therefore, the S protein may be the subject of future therapeutic inhibitor, antibody, and vaccination developments. According to Zhou et al. (2020), SARS-CoV-2 interacts with receptor ACE2. Blocking ACE2 to abrogate S protein binding may prevent the virus from attaching to host cells, thereby preventing viral entry [11,32,90].

*Veronica linariifolia* Pall. ex Link (Plantaginaceae) and *Rhus chinensis* var. *glabra* S.B.Liang (Anacardiaceae) were used to isolate tetra-O-galloyl-D-glucose (TGGl IC_50_ of 4.6 µM) and luteolin (IC_50_ of 10.6 µM) with an affinity for S2, blocking S2-mediated entry of viral particles into host cells. TGG exhibits a high selectivity index (SI; 24.0), indicating low toxicity. High SI values mean that the effects on an organism are less cytotoxic, which makes them very safe to use as antiviral agents in the future. The anthraquinone glycoside emodin, which was found in *Rheum officinale* Baill (Polygonaceae) and *Reynoutria multiflora* (Thunb.) Moldenke (Polygonaceae), significantly inhibited viral interactions between the ACE2 and S proteins [52]. An elderberry variety called *Sambucus formosana* Nakai (Viburnaceae) has also been found to exert an extremely strong antiviral impact due to its significantly high SI (154.37 g/mL) and extremely low IC50 (1.17 g/mL) against coronavirus [91]. For viral entry into a cell, the viral S protein leverages the host enzyme endosomal cathepsin L-protease [92,93]. By promoting receptor-mediated conformational changes mediated via the S2 domain and showing maximal proteolytic activity in the acidic environment of endosomes, cathepsin L increases the membrane fusion facilitated by the S protein. *Artemisia annua* f. *macrocephala* Pamp. (Asteraceae) chemical components, which were studied by Wang et al., 2007 using an in silico approach, have been reported to interact with endosomal cathepsin L-protease at a high negative binding energy (−50.767 kcal/mol) [94]. In the pathogenesis of the disease, clathrin-mediated endocytosis mediates SARS-CoV entry via the growth factor receptors transferrin receptor (TfR), keratinocyte growth factor receptor, and epidermal growth factor receptor. Butanol extraction of *Cinnamomum verum* J.Presl. (Lauraceae) Presl blocks clathrin-mediated endocytosis through the receptor TfR to treat wild-type SARS-CoV (wtSARSCoV) and HIV/SARS-CoV S pseudovirus infections [11].

### 3.3. Plants That Block Viral Replication and Translation

The ability of dietary flavanols to conjugate with the receptor ACE2 and inhibit the SARS-CoV-2 enzyme and protein activity, including chymotrypsin-like papain-like protease (PLpro), protease (3CLpro), (RdRp), and S protein activity, makes these compounds potential antiviral drugs [32,95].

Viral RNA and protein are required to support the life processes and pathogenesis of a virus. *Strobilanthes cusia* Kuntze (Acanthaceae), *Stephania tetrandra* S. Moore, (Menispermaceae), and *Sanguisorba officinalis* L. (Rosaceae), have been reported to inhibit viral RNA and protein synthesis. Moreover, natural alkaloids recovered from *Stephania tetrandra* S. Moore, such as fangchinoline, tetrandrine, and cepharanthine, have shown the ability to inhibit viral S and N protein synthesis [11].

*Sophora flavescens* Aiton (Fabaceae), *Torilis japonica* (Houtt.) DC (Apiaceae) and *Acanthopanax gracilistylus* W.W. (Araliaceae), similar to plant extracts, reduced not only RNA synthesis but also protein synthesis [58].

The functions of *Forsythia suspensa* (Thunb.) (Oleaceae) are frequently abrogated by RNA synthesis involving the viral N gene, emesis, pyrexia, and inflammation. RNA polymerase activity has also been reported to be inhibited by a number of plant extracts, including those obtained from *Coptis teeta* var. *chinensis* (Franch.) Finet & Gagnep. (Ranunculaceae), *Phoradendron meliae* Trel. (Santalaceae), and *Sophora subprostrata* Chun & H.Y.Chen (Fabaceae), *Phellodendron chinense* C.K.Schneid. (Rutaceae), and *Cimicifuga racemosa* (L.) Nutt. (Ranunculaceae) [59].

A transitional period of the COVID-19 outbreak was associated with the appearance of quickly proliferating SARS-CoV-2 mutations. Antivirals that target conserved genomic regions SARS-CoV-2, which are less likely to be subject to mutation, are urgently needed. The key element of a pathogen replication-transcription unit, “nsp12, commonly referred to as RdRp, is a potentially well-conserved therapeutic drug target. Certain RdRp nucleotide analogue inhibitors (NAIs)”, including remdesivir, have been reused with FDA approval to manage SARS-CoV-2 infection. The NAIs compete to prevent the insertion of a nucleotide into an RNA chain and target the translation of the RdRp protein, which prevents viral replication. The replication proofreading ability of nsp14-ExoN, however, might make SARS-CoV-2 resistant to several NAIs. “Nonnucleoside analogue inhibitors (NNAIs)”, on the other hand, attach to allosteric locations on the surface of the viral polymerase, affect the redox state, and subsequently exert antiviral activity by changing the interactions between the enzyme substrate and the active core catalytic region of RdRp. In contrast to NAIs, NNAIs do not require metabolic activation or compete with the intracellular pool of nucleotide triphosphates (NTPs) to inhibit RdRp activity. Compared to their synthetic counterparts, NNAIs derived from phytonutrients show prospective antiviral effects. A number of in silico studies have shown that natural phytonutrient NNAIs, such as silibinin (flavonolignan), suramin, theaflavin (tea polyphenol), lycorine (a pyrrolidine alkaloid), corilagin (a gallotannin), hesperidin (a citrus bioflavonoid), and baicalein (5,6,7-trihydroxyflavone), show a wider range of antiviral effects than favipiravir or remdesivir. These phytonutrient NNAIs exert multiple therapeutic effects in clinical COVID-19 therapy, including cardioprotective, antioxidant, and anti-inflammatory immunomodulatory effects [96]. Moreover, especially for nonnucleotide analogs, the fluorometric assay screening technique offers a useful foundation for the identification, validation, and assessment of new antiviral compounds targeting the SARS-CoV-2 RdRp [97]. The expression of active RdRp protein in *Escherichia coli* cells and baculovirus-infected insect cells was used to confirm the assay results. A reconstituted nsp12/nsp7/nsp8 complex and biotin-labeled self-priming RNAs were used to create an in vitro RdRp activity assay, and the activity of the RdRp complex was assessed by assessing RNA binding and extension. In *E. coli*, the functional RdRp holoenzymes nsp7 and nsp8 were identified and produced. Through an in vitro test to measure RdRp activity and a high-throughput screening strategy, the effects of the triphosphate form of remdesivir (RTP) and a number of other nonnucleotide analog viral polymerase inhibitors were found to be the same [97,98].

The main chemical, forsythoside A, an extract of *Forsythia suspensa*, was recently described by Shabana Bibi et al. to be a SARS-CoV-2 3CLpro inhibitor that obstructed viral multiplication and translation via a structural characterization. It is concluded that the target protein and lead chemical may help in the creation of future treatments for SARS-CoV-2 infection [99].

### 3.4. Inhibition of Viral Proteases 3CLpro and PLpro

Viral proteases such as 3CLpro and PLpro play significant roles in coronavirus replication, making them interesting therapeutic targets for the creation of anti-coronavirus drugs. SARS-CoV-2 3CLpro and PLpro cleave IRF3 are important modulators of the inflammatory pathway (NLRP12 and TAB1). To cleave host immune polyproteins during viral replication, the SARS-CoV-2 genetic machinery synthesizes two viral proteases, NSP5/3C-like protease and NSP3/papain-like protease [100].

In 2021, Yongzhen Liu et al. provided a fresh perspective for understanding SARS-CoV-2 immune evasion tactics and suggested prospective antiviral medications to treat people with CoV disease 2019 [77]. The major SARS-CoV-2 protease Nsp5 negates RIG-I and the mitochondrial antiviral signaling (MAVS) protein activity in two different ways. Specifically, Nsp5 prevents RIG-I from activating MAVS by cleaving the 10 most-terminal amino acids in the N-terminus, and Nsp5 promotes the ubiquitination and proteasome-mediated destruction of MAVS. Therefore, depending on the targeted enzyme, Nsp5 strongly prevents double-stranded RNA from inducing interferon (IFN) production. An artificial small-molecule inhibitor prevents the processing of SARS-CoV-2 non-structural proteins and the destruction of cellular RIG-I and MAVS caused by Nsp5. This effect restores the innate immune response and prevents the spread of SARS-CoV-2 [77].

A novel method by which the SARS-CoV and SARS-CoV-2 NSP5 protein inhibit the synthesis of IFN involves preventing phosphorylation of IRF3 in the cytoplasm. This finding has implications for the development of effective antiviral drugs to combat SARS-CoV-2 [101].

The interaction energies obtained by docking the quercetin inhibitor with its targets PLpro and 3CLpro were −4.62 and −6.25 kcal/mol, respectively. The potential for quercetin to prevent SARS-CoV-2 replication is currently largely theoretical [102].

To stop the COVID-19 pandemic, antiviral medicines that prevent SARS-CoV-2 replication are urgently needed, in addition to vaccines. Papain-like protease (3CLpro) and 3C-like protease (3CL) are the only viral cysteine proteases required for viral replication and assembly (PLpro). The deubiquitinase (DUB) activity of PLpro, which removes ubiquitin (Ub) and Ub-like modifications from host proteins, slows the immune response of a host. Novel noncovalent SARS-CoV-2 PLpro inhibitors based on 2-phenylthiophene have been described by Zhengnan Shen et al. to block viral replication in monkey and human cell cultured cells, controlling this complex process [103]. According to in silico-based research conducted by Satyam Singh, hesperidin, theaflavin, theaflavin-3′-O-gallate (TF2a), (TF1), theaflavin-3′-digallate (TF3), theaflavin-3′-gallate (TF2b), myricetin, and quercetagetin all showed a substantial affinity for the RdRp active site [104].

The highest docking affinities were observed for PLpro (−8.8 kcal/mol) with gallocatechin gallate and 3CLpro (−9.4 kcal/mol) with amentoflavone. Other phytochemicals with high affinity included kazinol A, theaflavin-3,3-digallate, and savinin [105]. Tomatidine, in particular, and patchouli alcohol may offer viable treatments for SARS-CoV-2 infections by preventing viral replication [106].

Recently, Gupta et al. conducted studies on seven proteinaceous molecules, including PLpro and 3CLpro, whose enzymatic functions were found to be crucial at multiple levels of the viral cycle. Bisindolylmaleimide IX has been reported to inhibit 3CLpro protease [107]. The in vitro inhibitory effects of sodium fluoride, hexetidine, menthol, eucalyptol, triclosan, chlorhexidine, and aloin A and B have been evaluated against viral proteases 3CLpro and PLpro, and aloins A and B were the only ones found to suppress the protein degradation activity of PLpro, with observed IC_50_ values of 13.16 and 16.08 M, respectively. Aloin A and B isomers also showed deubiquitination inhibitory activities with IC_50_ values of 15.68 and 17.51 M, respectively. The isoforms of aloin limited SARS-CoV-2 PLpro proteolytic and deubiquitinating activities, indicating their possible use in preventing viral reproduction [108].

Various computational approaches have been utilized to identify a large number of plants with compounds that show binding interactions with SARS-CoV-2. However, the results obtained from computational studies corroborated in vitro and in vivo experiments and inspired the traditional use of various phytodrugs. Deng-Hai Zhang et al. used a computational approach to identify the antiviral potential of various orally administered Chinese herbal compounds, namely, betulinic acid, coumaroyltyramine, cryptotanshinone, desmethoxyreserpine, dihomo-c-linolenic acid, kaempferol, dihydrotanshinone, kaempferol, moupinamide, lignan, quercetin, N-cis-feruloyltyramine, and tanshinone IIa. These molecules inhibited SARS-CoV-2 infectivity by targeting PLpro and 3CLpro [109,110].

The potential inhibitors of SARS-CoV-2 Mpro protease activity that potentiate infection have been identified via computational approaches. These plants with inhibitory products were *Withania somnifera* (L.) Dunal: Ashwagandha (Solanaceae), *Ocimum sanctum*: Tulsi (Lamiaceae), and *Tinospora cordifolia* (Willd.) Miers ex Hook.f. & Thomson: Giloy (Menispermaceae); their different chemical components included somniferine, with a binding energy of −9.62 kcal/mol; withanoside V, with a binding energy of −10.32 kcal/mol; isoorientin 40-O-glucoside 200-O-p-hydroxy benzoate, with a binding energy of −8.55 kcal/mol; vicenin, with a binding energy of −8.97 kcal/mol; ursolic acid, with a binding energy of −8.52 kcal/mol; and tinocordiside, with a binding energy of −8.10 kcal/mol [111]. In a computational study, a library of more than 100 steroidal plant-derived pregnanes (PDPs) were associated with the target active site of human glucocorticoid receptors (hGRs). In this study, 20 molecules were found to show significant activity against the target with a binding energy range from −9.8 to −11.2 kcal/mol. These PDPs were then examined to ascertain their potential interactions with human Janus kinases 1 and interleukin-6 as well as SARS-CoV-2 3-chymotrypsin-like protease [112].

### 3.5. Plants and Furin-Like Proteases

A virtual screening revealed furin-like proteases disrupted spike protein-related virus maturation and therefore may be a target to control COVID-19. The results of this study showed that active herbal chemical compounds, such as xanthones from phyllaemblicin G7, biorobin, and andrographolide, including their derivatives, potentially inhibited furin. The pharmacological potential of *Withania somnifera* (L.) Dunal (Solanaceae), popularly known as *Ashwagandha*, has been extensively explored. Recently, computational studies on active constituents revealed that withanone blocked electrostatic interactions and destroyed the salt bridge critical for facilitating the interaction between host virus-binding proteins and viral ACE2 receptors. Thus, withanone prevented the entry of the virus into the host cell and may be a drug to manage COVID-19 [109,113].

Screening of certain plant-derived bioactive molecules such as Mpro inhibitors to ascertain their effects against main proteases was conducted by molecular docking. Quercetin, kaempferol luteolin-7-glucoside, naringenin, demethoxycurcumin apigenin-7-glucoside, curcumin, oleuropein epicatechin-gallate, and catechin were reported to show significant binding interactions and thus may be COVID-19 Mpro inhibitors. However, more studies are required to examine their effectiveness for therapeutic use [51]. The pharmacological activity of *Nigella sativa* L. (Ranunculaceae) has been extensively explored. Molecular docking with its active constituents against SARS-CoV-2 suggested that nigellidine and α-hederin inhibited the viral proteases 3CLpro/Mpro more significantly than the other compounds analyzed.

In addition to molecular docking, the use of artificial intelligence (AI) has markedly increased in the past two years of the COVID-19 pandemic, especially in the field of drug discovery as led by giant pharmaceutical companies. One study explored the use of AI by the pharmaceutical industry for different purposes, such as drug repurposing and reducing the time for clinical trials [114]. Due to clinical trial processes and the approval of new treatment plans or drugs/vaccines, drug development is a lengthy and time-consuming process. Hence, by utilizing modern algorithms that are properly supported by highly configured hardware, new AI-based technologies have been proven to be the most valuable tools in drug discovery [115].

A combination of AI and traditional drug design methods were used to categorize and identify anti-COVID-19 inhibitors from a library of 125 natural compounds that can target the spike protein of SARS-CoV-2 [116]. During the COVID-19 pandemic, AI supported the rapid development of inhibitor molecules extracted from or abundant in plants that had never been screened to determine their therapeutic effect against SARS-CoV-2 [117].

Interestingly, the SARS-CoV-2 viral sequence was identified by AI, and a model generated via AI may be helpful in the identification of the delta and omicron strains [118]. Several AI-based models have been developed and extensively trained for the quick and high-throughput testing and screening of natural compound databases. KC et al. developed REDIAL-2020, a web-based drug molecule discovery tool through which eleven AI models can be utilized for identifying highly active compounds against SARS-CoV-2 [119]. CoronaDB-AI is a database training model containing a library of peptides, epitopes, and chemical structures. This deep learning-based model is being utilized for immediate support in viral disease treatment discoveries [120].

## 4. Plants as Biological Factories for the Production of Immunotherapeutics: Applications to SARS-CoV

Various immunomodulators, antibodies, interferons, and other therapeutic proteins have been used in the management of various diseases, including virus-induced diseases. Clinical trials for treatments of MERS and SARS-CoV have revealed the role of interferon in reducing the severity of the diseases caused by these coronaviruses.

Plants have been used as biological factories for the production of immunotherapeutics. Various plant-derived vaccines have been assessed in clinical trials, and a few are now marketed as medications and in conjunction with medical devices for the treatment of infectious and chronic diseases. Plant-based vaccines might enable the rapid production of biological products on an industrial scale, which may help meet needs in urgent situations such as the COVID-19 epidemic. Some vaccinations, including those used for cholera, anthrax, Lyme disease, tetanus, rotavirus, canine parvovirus, and plague, were created through direct particle bombardment or biological means [121]. Vaccines for Ebola, tuberculosis, avian flu, and dengue fever are manufactured indirectly or directly via Agrobacterium-mediated gene transfer [121,122].

One study demonstrated that IFN-α inhibited the replication of human- as well as animal-infecting coronaviruses [123]. Another study reported that interferon-α-2a administered with ribavirin increased survival in patients with MERS-CoV [124]. The very first recombinant medicinal protein made from a plant was human interferon, which was created in turnips. Moreover, tobacco plants and potatoes have been used to manufacture human serum albumin for human use. Similarly, tobacco plants were used to generate the first medication (ZMapp) used experimentally to treat Ebola virus infection.

Intravenous gamma globulin (InIg) has also been studied. It was developed in 1970 but gained popularity during the outbreak of SARS in 2003, when it was used extensively in Singapore. However, severe adverse reactions were noticed during its use [125]. Scientists have developed methods for the production of gamma globulin using plant-based molecular farming.

A peptide hormone, thymosin-α-1, has been isolated from thymic tissues, and its immunomodulatory role has been thoroughly explored [126]. Moreover, a synthetic pentapeptide such as thymopentin interacting with the active site in thymopoietin has been found to boost the production of antibodies for hepatitis B vaccines [127]. Plant bioreactors have been successfully applied to the production of thymosin-α-1 [128,129].

Due to the development and expansion of recombinant techniques, plants are now being assessed as potential alternative platforms for the manufacture of recombinant monoclonal antibodies (mAbs). In a study by Sui et al., r-mAb, a recombinant human monoclonal antibody against the S1 domain of the S protein of SARS-CoV was isolated. Moreover, this mAb had been found to efficiently neutralize SARS-CoV [130]. Recent research demonstrated that plant systems for producing mAbs for immunotherapy have been successfully developed [131].

The immunotherapeutics used as the basis for developing SARS-CoV-2 vaccines are thought to be potentially effective and safe. When considering the potential delivery of SARS-CoV-2 vaccines in the future, current guidelines for immunizing a host must be used [132].

For the first time, the recombinant production of a fully functional human form of the recently identified cytokine IL-37 in plant cells has been reported, with the cytokine functioning as a fundamental suppressor of innate immunity. Interleukin 37 (IL-37), a recently identified member in the interleukin (IL)-1 class, is essential for controlling innate inflammation and inhibiting acquired immunological responses and, therefore, it shows great potential for treating a variety of autoimmune diseases and inflammatory diseases [133]. Recombinant IL-7 is a potential immune therapeutic that acts to promote the proliferation of naive and memory T cells (CD8+ and CD4+ T cells) and may be effective in managing SARS-CoV-2 viral infection. IL-6 is a key mediator of inflammation in COVID-19, and its receptor antagonist (sarilumab/tocilizumab) and IL-6 inhibitors (clazakizumab/siltuximab/sirukumab) have been described as potential immunotherapeutics to manage SARS-CoV-2 viral infections [134].

The IL-1 inhibitors canakinumab (MoAb anti-IL-1beta) and anakinra (recombinant IL-1 receptor) inhibited IL-1β, a proinflammatory cytokine [135,136]. Other significant immunotherapeutic IL-1 inhibitors, such as canakinumab (MoAb anti-IL-1beta) and anakinra (a recombinant IL-1 receptor), as well as IL-18 inhibitors, have been reported to inhibit IL-1β. Suppression of signaling that triggers the cytokine stimulation of cytokines such as IL-7 and alters Type I IFN levels significantly enhanced the risk of thromboembolism after treatment with JAK/STAT inhibitors such as ruxolitinib, baricitinib, and tofacitinib [137]. Anti-VEGF, complement factor C3, C5, and complement system inhibitors are other classes of immune therapeutics that may be effective against COVID-19. Bamlanivimab antibodies have also been described as being effective against the spike protein of SARS-CoV-2. Tomato plants have been examined for use in the development of SARS vaccines; specifically, their ability to express SARS-CoV nucleocapsid proteins, and their immunogenicity for the development of vaccines have been assessed. A COVID-19 vaccine is now being developed by the Kentucky Bio-Processing Company, a British American Tobacco (BAT) subsidiary, using tobacco plants to express the SARS-CoV-2 protein subunit. The receptor binding protein or the sequence of the S1 protein (full polypeptide) may be the intended vaccine target [72]. Hence, these findings indicate that plants have enormous promise for the low-cost and high production of biologically active viral inhibitors.

### 4.1. Drugs and Chemicals

In a study conducted by Joffe et al., levamisole, a new synthetic drug given along with ascorbic acid to measles patients, was found to increase the lymphocyte subpopulation. Ascorbic acid was also tested in chick embryo tracheal organ cultures, and after experimentation, resistance to coronavirus was observed [138]. Cyclosporine A is an immunosuppressive drug that significantly enhanced the persistence rates of patients as well as the success of organ transplantation [139]. Cyclosporine A was observed to bind a principle immunophilin, namely, cyclophilin, which binds to the nucleocapsid protein of SARS-CoV. Hence, inhibition of cyclophilin A by cyclosporine A can block the replication of all species of coronaviruses, including SARS-CoV [140]. Chloroquine and hydroxychloroquine are both antimalarial agents. Experiments have shown that these two treatments can potentially inhibit the replication of several microorganisms, including human coronaviruses. Both drugs render the endosomal pH basic and thus interfere with the glycosylation of the cellular receptor of SARS-CoV, preventing viral infection [141].

Previous studies with SARS-CoV-1 demonstrated that chloroquine and hydroxychloroquine caused a deficit in the glycosylation receptors at the virus surface such that the viruses could not bind to the receptor angiotensin-converting enzyme-2 expressed in the lungs, kidney, heart, and intestine. Since the same mechanism for binding to the cell surface is utilized by SARS-CoV-2, it is believed that chloroquine and hydroxychloroquine can inhibit the attachment of this virus to target cells [142].

Clinical reports suggested that azithromycin given with hydroxychloroquine showed a synergistic effect. Azithromycin has been shown to be active in vitro against Ebola and Zika [143,144]. Promazine is an antipsychotic drug that has shown a potential inhibitory effect on the replication of SARS-CoV. It blocks the interaction of the S protein with the receptor ACE-2 [145]. Recently, surface plasmon resonance (SPR) has been used to identify viral membrane protein ligands and target active ingredients. SPR was performed with senkyunolide, an active constituent of *Chuanxiong* extract, and the chemokine receptor 4 C-X-C, also known as the CXCR4 receptor protein for the virus. The affinity constant of 2.94 ± 0.36 μM suggested an interaction between senkyunolide I and CXCR4. Further analysis with a Boyden chamber assay revealed that senkyunolide I hindered the movement of cells. Thus, SPR-based screening via a viral membrane protein-targeting active ligand interaction strategy showed that senkyunolide may be effective in managing COVID-19 [145]. In vitro research reported that the ritonavir–lopinavir combination exerted a significant effect against SARS-CoV-2 at the micromolecular IC-50 level. In a different clinical trial study, other combinations, ribavirin, interferon-beta-1b, and lopinavir–ritonavir, were compared to ritonavir–lopinavir treatment alone (control arm), and reported higher safety and potency with decreasing symptoms, shortened duration of viral shielding, and reduced hospital stays in patients with mild-to-moderate *hepatitis C*. COVID-19 [146]. Umifenovir, a second broad-spectrum antiviral that has been approved in Russia for influenza treatment, was also discovered to have activity against coronaviruses as tested in virus-infected cell culture assays, umifenovir prevented membrane attachment of SARS-CoV-2 to host cells [147]. Umifenovir combined with lopinavir/ritonavir is currently being compared to the combined form of this medicine administered with interferon in a multicentre randomized controlled trial (ChiCTR2000029573) to determine which combination is most effective [148,149].

### 4.2. Roles of Plant Dietary Supplements

Plants are excellent sources of antioxidants, anti-inflammatory molecules, minerals, vitamins, fats, and proteins that play significant roles in the wellbeing of patients. Because of the potential to lower the risk of infection while simultaneously enhancing the health of COVID-19 patients, special consideration should be given to nutrients that play significant roles in the control of the immune response. Vitamins C, D, and zinc are micronutrients for which the evidence is strongest for their support of the immune system [150].

Nutritional factors that can boost immunity in persons suffering from COVID-19 are listed herein. These elements not only increase immune responses but also act as antioxidants. The roles of water-soluble vitamins such as vitamin B found in legumes (pulses, such as beans), spinach, asparagus, whole grains, potatoes, chili peppers, bananas, and breakfast cereals; vitamin C in citrus fruits involved in collagen synthesis; and antioxidants, have been established. Since these nutrients help to boost immunity, they may be used to combat coronavirus infection. Clinical studies of vitamin C supplementation in pneumonia patients showed that vitamin C clearly reduced the frequency of pneumonia development. Thus, we suggest that vitamin C might reduce the likelihood of respiratory tract infections under certain conditions [151,152].

Vitamin D, which is naturally obtained from mushrooms, is a fat-soluble vitamin that helps to maintain bone integrity and stimulates the growth of different types of cells, including immune cells. Clinical studies have reported that vitamin D deficiency may cause bovine coronavirus infection [153,154].

Fat-soluble vitamin E is found in wheat germ oil, almond, sunflower seeds, pine nuts, avocados, red bell peppers, etc., and plays an important role in oxidative stress. Previously studies with mice reported an increase in the virulence of coxsackievirus B3 due to a deficiency in vitamin E [151,152]. Lipid-soluble vitamin A is an anti-infectivity factor. Many immune diseases have been reported due to a lack of vitamin A; these diseases include measles and HIV. Moreover, the potentiation of bronchitis after coronavirus infection has been reported in a study conducted with chickens fed a diet with a lower vitamin A content and chickens fed a diet with a higher vitamin A content. Long-chain polyunsaturated fatty omega 3 fatty acid has been reported to be important. Omega-3 polyunsaturated fatty acids are important facilitators of adaptive immune responses, particularly under inflammatory conditions [151,155,156].

Zinc is an important dietary mineral found in many grains, such as maize, barley, sunflowers, vegetables, and fruit, and it is used for developing and maintaining immune cells for both adaptive immunity and innate immunity. Zinc deficiency results in the malfunctioning of both cell-mediated and humoral immunity and increases vulnerability to infectious diseases. The combination of zinc with pyrithione has been shown to inhibit the replication of COVID-19 [136,157,158]. Studies have shown that dietary deficiency of selenium produces alterations in the viral genome under oxidative stress that result in the potentiation of viral pathogenesis. Therefore, selenium intake may be a good choice for the control of novel infectious coronaviruses [159]. Iron deficiency has been reported to be a risk factor in recurrent acute respiratory tract infections. Nutritional factors that can help boost immunity in persons suffering from COVID-19 are listed herein. These elements not only increase immune responses but also act as antioxidants. Moreover, nutritional components, such as vitamins, can control not only pathological processes but also many physiological processes and directly affect the epigenome [160,161]. The number of reports on botanical medicines and dietary supplements as sources of possible therapeutic agents for SARS-CoV-2 medication development is increasing [162]. Research is required to determine the mechanisms of action and efficacy of phytotherapeutic interventions when used as adjunctive drugs during the onset or recovery of SARS-CoV-2 exposure, with or without a vaccine. Moreover, we have good reason to expressly caution against the promotion of treatments without supporting data and against grossly deceptive or plainly incorrect claims [150].

## 5. Conclusions

The medicinal plant resources that may be effective in the treatment of COVID-19 are claimed on the basis of the history of using these herbs in the treatment of MERS and SARS. In addition, other medicinal herbs have been identified worldwide that may play a beneficial role in combatting COVID-19. As the virus continues to spread at a very high rate, the main focus is the development of a therapy that can prevent the entry of the virus into the host. These herbs may contribute to this inhibitory effect by increasing the effectiveness of the host immune response. Since some countries have developed vaccines for the disease, the cost of which may be high, we must develop alternate inexpensive sources of drugs to treat COVID-19 patients in developing countries. To this end, medicinal plants can play a significant role. New therapy options for SARS-CoV-2 are required, even though different vaccines are being produced, because plant-derived chemicals show a wide spectrum of therapeutic actions, particularly against SARS-CoV-2, making them advantageous on SARS-CoV-2 targets. Plants such as *Glycyrrhiza glabra*, *Andrographis paniculata*, *Azadirachta indica*, *Curcuma longa*, *Ocimum sanctum*, *Withania somnifera*, *Allium sativum*, *Zingiber officinale*, *Moringa oleifera*, *Tinospora cordifolia*, and *Nigella sativa* have been reported to exhibit significant antiviral activity against SARS-CoV-2. Moreover, the in vitro activity of many plants or derived molecules including umifenovir, hydroxychloroquine and chloroquine, glycyrrhizin, turmeric (*Curcuma longa*) rhizomes, mustard (*Brassica nigra*), and wall rocket (*Diplotaxis erucoides* subsp. *erucoides*), were tested effectively against corona viruses. The potential for using the majority of plant-derived bioactive compounds is increased because clinical studies of other infectious diseases have confirmed their effectiveness and safety in the human body. The molecules must be evaluated specifically against SARS-CoV-2, as the majority of the trials chosen for this study did not specifically assess how direct chemical treatment affected the virus. Therefore, when prescribing natural compounds, extreme caution is needed. Thus, future studies must start, and they must intervene in a suitable dosage form.

## Data Availability

Not applicable.

## Supplementary Materials

The following supporting information can be downloaded from https://www.mdpi.com/article/10.3390/ijms232113564/s1.
