# Supplementary material for: Antiviral Potential of Plants against COVID-19 during Outbreaks—An Update
Source: Int J Mol Sci. 2022 Nov 5;23(21):13564. doi: 10.3390/ijms232113564 (PMC9655040; doi:10.3390/ijms232113564)
Supplement: Supplementary file 1 [file ijms-23-13564-s001.zip › ijms-1863344-supplementary.pdf]

Table S1. Antiviral potential of Medicinal Plants

Common name (Traditional Asian)	Botanical name & parts of the plant	Chemical constituents	Effective in Viral disease	Reference
Lahsun	*Allium cepa, bulb*	Allicin, E/Z-ajoene, Allin, Diallyl disulfide, Diallyl trisulfide, pyrogallol, protocatechuic acid, Quercetin, gallic acid.	COVID19,Influenza virus	[41]
Kali mirch	*Piper longum, fruits*	α-pinene, β-pinene, limonene, myrcene, sabinene, camphene, α-tujone, piperitone, caryophyllene, p-cymene, α-terpinene, piperamide.	Coxsackie virus type-3 (CVB3),	[44]
Haldi	*Curcuma longa, rhizome*	Curcumin, curcumenone, bisacumol, bisacurone, Curcumenol, curcumadiol, demethoxycurcumin.	COVID19, Zika virus, Chickungunya virus, vesicular stomatitis virus	[46]
Clove pink	*Dianthus caryophyllus,root, flower*	Dianthin30, dianthin32,dianthramides,flavonoid	COVID19, Herpes simplex virus-I(HSV-I),Hepatitis-A Virus-27(HSV-27)	[44-45]
Laung	*Syzygium aromaticum, flowering buds*	Eugenol, acetyl eugenol, β-caryophyllene, vanillin, eugenin, kaempferol, Rhamnetin, eugenitin	COVID19, Herpes simplex virus-I, Herpes simplex virus-2, Hepatitis C virus	[47]
Adrak	*Ginger officinalis, rhizome*	6-Gingerol, 6-Shogaol, 6-Paradol, Zingerol, Gingerol.	COVID19, Human respiratory syncytial virus (HSRV)	[51]
Garden Rhubarb	*Rheum rhabarbarum, whole plant*	Emodin	SARS-CoV	[52]
Dalchini	*Cinnamomum zeylanicum, bark*	Cinnamaldehyde, Eugenol, cinnamic acid, cinnamyl acetate, cinnamyl alcohol,Thujene	Immunostimulatory action	[53]
Pokeweed	*Phytolacca Americana,root, Brries&leaves*	Phytolaccoside-A,B Phytolacca saponine-B, Phytolaccagenic acid, pokeberrygenin, hederogenin	HIV-I, Human T-cell Leukemia virus	[54]
Four o'clock flower	*Mirabilis jalapa,wholeplant*	4-hydroxycoumarin,MirabijaloneA-D, 9-o-methyl-4-hydroxyboeravinoneB,Campesterol	HIV-I	[55]

Green tea	*Camellia sinensis, leaves*	(-)-epigallocatechin gallate,(-)-epigallocatechin, (-)-epicatechingallate, (-)-epicatechin,(+)-catechin	COVID19	[51,56,57]
Chaihu	*Radix Bupleuri, roots*	Saikosaponins A, B_2,C and d	HCoV-22E9	[67,73,74]
Liquorice root	*Glycyrrhiza glabra, roots*	Glycyrrhizin Glycyrrhizic acid	SARSr virus	[68,75]
Chinese skullcap	*Radix Scutellaria*	Baicalin	SARS-CoV	[76-77]
Fish mint	*Houttuynia cordata, leaves*	Cinanserin	3CLpro inhibitors of SARS-CoV, covid-19	[78-79]
Japanese alder	*Alnus japonica,stem bark, root*	Diarylheptanoids	PLP inhibitors of SARS-CoV	[80,81,82]
Tobacco	*Nicotiana tabacum, leaves*	Nicotianamine	ACE-2 inhibitor in SRAS-CoV	[85]
Citrus fruit	*Citrus sinensis, fruits*	Naringenin	Inhibits Mpro of SARS-CoV	10.1016/j.jsps.2022.02.014[51]
olive	*Olea Europaea, fruits*	Apigenine-7-glucoside, Oleuropein	Inhibits Mpro of SARS-CoV	10.1080/21655979.2022.2027181 [51]
Chili pepper	*Capsicum annum, fruits*	Quercetin, Luteoline-7-glucoside	SARS-CoV	[69-70]
Boswellia	*Extract*		Viral infection	[72]
Isobavachalcone,	*Rhoifolin Pectolinarin*	Quercetin 3-β-d glucoside, Helichrysetin	SARS-CoV	[60-70;102]
**Nutritional element**				
**Name**	**Dietary/Other source**	**Benefits offered**	**Effective in Viral disease**	**Ref**
Vitamin C	Amla, Lemon, Oranges	An immune booster	Antiviral, Anticancer	[51-52]
Vitamin D	Soy milk, Beef Liver, Cheese, Egg Yolks	Raise the resistance level	Antiviral,COVID-19	[153.154]
Vitamin E	Soybean oil, Wheat Germ oil, Rice Germ oil	Reduce oxidative stress	Antiviral, Anticancer	[153.154]
Vitamin A	Carrot, Spinach, Papaya, Mango	Raise innate immunity	Antiviral, Anticancer	155,156,151
Omega-3-polyunsaturated fatty acid	Cereals, vegetables, and nuts	Raise the levels of prostaglandin/leukotriene/protectine	Omega-3-polyunsaturated fatty acid which is commonly found in Cereals, vegetables, and nuts has been reported to raise the levels of prostaglandin/leukotriene	[153.154]

			/protectine in iAntiviral, Anticancer protective wayAntiviral, Anticancer	
Zinc	mushrooms, spinach, broccoli, kale, and garlic	Raise the level of humoral & cell mediated immunity	Antiviral, Anticancer	[151,159]
Selenium	Cereals ,Jaggery, Ragi	Increases the host immune system	Antiviral, Anticancer	[151,160]
Iron	Legumes, Cereals, Green leafy vegetables, Fruits	Prevents development of recurrent acute respiratory tract infection	Antiviral, Anticancer	[161-162]
